# Supplementary material for: ern: An R package to estimate the effective reproduction number using clinical and wastewater surveillance data
Source: PLoS One. 2024 Jun 21;19(6):e0305550. doi: 10.1371/journal.pone.0305550 (PMC11192340; doi:10.1371/journal.pone.0305550)
Supplement: S1 File — (PDF) [file pone.0305550.s001.pdf]

SUPPLEMENTARY MATERIAL - S1

-

`ern`: an R package to estimate the effective reproduction number using clinical and wastewater surveillance data

David Champredon[1*], Irena Papst[1], Warsame Yusuf[1]

1 Public Health Risk Sciences Division, Public Health Agency of Canada, National Microbiology Laboratory, Guelph, Ontario, Canada

* david.champredon@canada.ca

Methodological differences when inferring daily incidence Inferences of daily incidence from aggregated clinical reports depend on the assumptions made (explicitly or implicitly). Here, we briefly illustrate the potential differences in $\mathcal{R}_t$ inferences between the methods implemented in `ern` (*i.e.,* transmission model based on the renewal equation and linear interpolation) and the method implemented in the R package `EpiEstim`, which uses an exponential interpolation technique. The example presented below simply aims at warning the reader that the choice of disaggregation method may have a significant impact on $\mathcal{R}_t$ estimates. Making a thorough comparison of the different methods is beyond the scope of this article. The code to perform this simple example is provided in supplementary file S7.

We aggregated the daily "SARS2003" data attached to the `EpiEstim` package to produce a weekly clinical data set spanning two months. Next, we set up a common set of $\mathcal{R}_t$ estimation settings across the `ern` and `EpiEstim` packages: we used the same generation interval distribution (a Gamma distribution with mean 3.5 days), a sliding window of 7 days, reporting delay and incubation period close to 0 days (because as `EpiEstim` does not handle those explicitly and they cannot be ignored in `ern`). Then, we run the $\mathcal{R}_t$ inferences for both packages, and additionally retrieved the inferred daily incidence from each method. The results are shown in Figure S1. We see that the different disaggregation methods can lead to markedly different $\mathcal{R}_t$ estimates (top panel) and inferred daily incidence (bottom panel). In this example, none of the methods can infer the "true" daily incidence (Figure S1, bottom panel, blue curve) Again, we note that this example is simply an illustration of the differences between various methods, not a systematic methodological comparison.

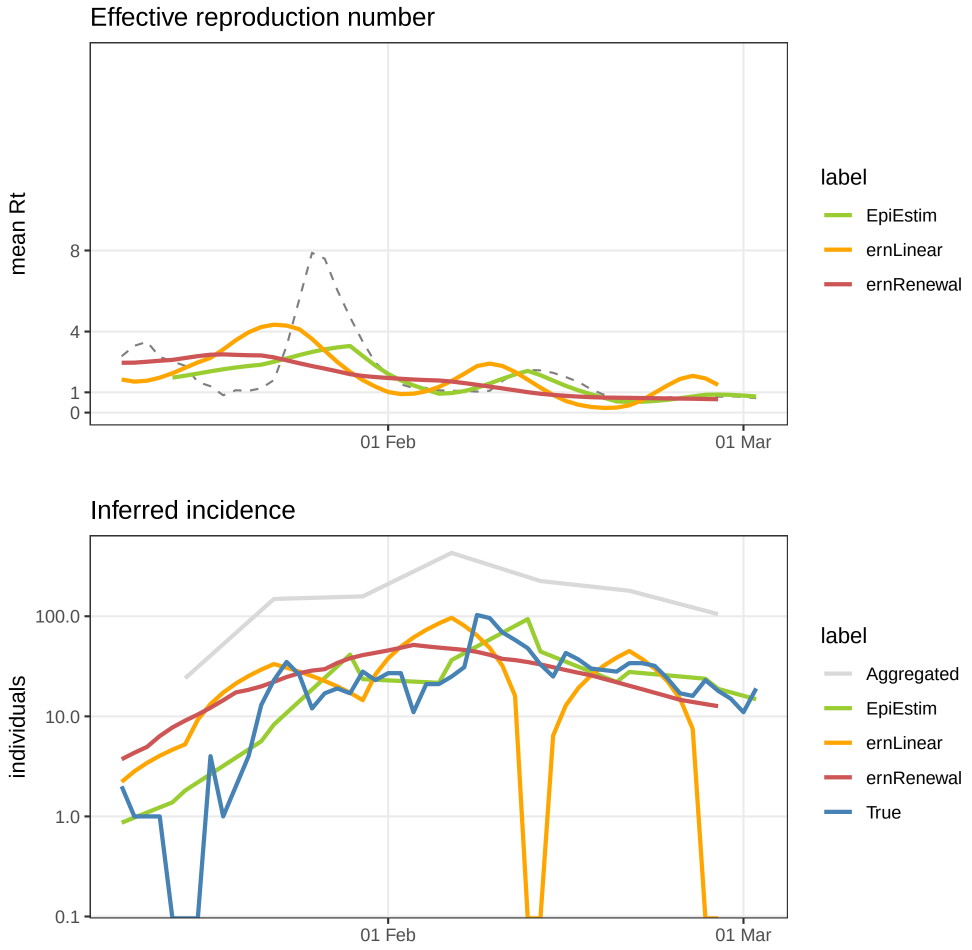

Fig S1. Impact of the methodology used to interpolate daily incidence on $\mathcal{R}_t$. Top panel: mean $\mathcal{R}_t$ estimates from `ern` and `EpiEstim`. Grey dashed line: mean $\mathcal{R}_t$ estimates from `ern` using daily data. Bottom panel: True daily incidence (blue curve) and aggregated weekly incidence (grey curve) on the log scale. Coloured curves represent the mean estimates of daily incidence from `ern` and `EpiEstim` (see legend)
