# Supplementary material for: ern: An R package to estimate the effective reproduction number using clinical and wastewater surveillance data
Source: PLoS One. 2024 Jun 21;19(6):e0305550. doi: 10.1371/journal.pone.0305550 (PMC11192340; doi:10.1371/journal.pone.0305550)
Supplement: S2 File — (PDF) [file pone.0305550.s002.pdf]

SUPPLEMENTARY MATERIAL - S2

-

`ern`: an R package to estimate the effective reproduction number using clinical and wastewater surveillance data

David Champredon[1*], Irena Papst[1], Warsame Yusuf[1]

1 Public Health Risk Sciences Division, Public Health Agency of Canada, National Microbiology Laboratory, Guelph, Ontario, Canada

* david.champredon@canada.ca

Bayesian model to infer daily clinical report count Let $i(t)$ be the true incidence on day t, $A(t)$ the sum of the daily (true) incidence over the past ℓ days (*i.e.,* the aggregated weekly incidence), and $Y(t)$ the observed cases aggregated over the past ℓ days. The package `ern` applies the model described below to interpolate the *reported* cases Y.

The number of individuals still susceptible to infection at time t is denoted $S(t)$ and the basic reproduction number (the average number of secondary infections caused by the introduction of one infectious person in a *fully* susceptible population) is $\mathcal{R}_0$. Finally, we denote the intrinsic generation interval distribution as g. We assume the epidemic is driven by the renewal equation:

$$i(t) = \mathcal{R}_0 \left(\frac{S_t}{N}\right)^{1+\alpha} \sum_{k=1}^{t} i(t-k)g(k) \tag{S1}$$

This is equivalent to the classical "Susceptible-Infected-Recovered" (SIR) epidemic process modelled with a system of ordinary differential equations [**?**]. The parameter α represents the level of heterogeneity of transmission in the population, with $\alpha = 0$ corresponding to homogeneous mixing [**?**]. The depletion of susceptible individuals is modelled as:

$$S(t) = \max(0, S(t-1) - i(t)) \tag{S2}$$

We assume that daily incidence cannot be observed directly, but that the cumulative sum of incidence over some time period is observed (typically these are weekly aggregates). We define the aggregated incidence as

$$A(t) = \sum_{k=0}^{\ell} i(t-k) \tag{S3}$$

where ℓ is the aggregation window (*e.g.,* $\ell = 7$ for weekly aggregation). Note that `ern`'s implementation allows for a variable aggregation window (*i.e.,* ℓ does not have to be constant). Since $Y(t)$ is the *observed* aggregated number of cases at time t, we assume it follows a Poisson distribution to account for observation noise:

$$Y(t) \sim \text{Poisson}(A(t)) \tag{S4}$$

Note that we do not assume under- or over-reporting, as we simply want to interpolate incidence for the missing dates. In fitting the model, we assume the following priors:

$$\mathcal{R}_0 \sim \text{Gamma}(2, 0.6) \tag{S5}$$

$$\alpha \sim \text{Gamma}(1, 1) \tag{S6}$$

Here we use the shape (first input parameter) and rate parameterization of the Gamma distribution. Hence we have a hierarchical model with latent variables.

Given the observed aggregated cases time series $Y(t)$, the latent daily incidence time series $i(t)$ is estimated by inferring the hyper-parameters $\mathcal{R}_0$ and α using a Markov Chain Monte Carlo algorithm. We use the R library `rjags` to run this inference [?]. The posterior realizations of $i(t)$ define the ensemble of daily incidence, whose individual realizations are repeatedly taken as input into the function that estimates the effective reproduction number $\mathcal{R}_t$, in order to produce an ensemble of $\mathcal{R}_t$ (among other resamplings of the relevant families of distributions, like the incubation period, reporting delay, reporting fraction, and intrinsic generation interval).

This method for interpolating aggregate case reports is specified using the argument `prm.daily = list(method = "renewal")` in the function `ern::estimate_R_cl()`.
