# Supplementary material for: ern: An R package to estimate the effective reproduction number using clinical and wastewater surveillance data
Source: PLoS One. 2024 Jun 21;19(6):e0305550. doi: 10.1371/journal.pone.0305550 (PMC11192340; doi:10.1371/journal.pone.0305550)
Supplement: S3 File — (PDF) [file pone.0305550.s003.pdf]

SUPPLEMENTARY MATERIAL - S3

-

`ern`: an R package to estimate the effective reproduction number using clinical and wastewater surveillance data

David Champredon[1*], Irena Papst[1], Warsame Yusuf[1]

1 Public Health Risk Sciences Division, Public Health Agency of Canada, National Microbiology Laboratory, Guelph, Ontario, Canada

* david.champredon@canada.ca

Linear interpolation to infer daily clinical report count Let $A(t)$ be the aggregated count of clinical reports at time t, $\tau(k)$ the kth aggregation time and $y(t)$ the (unknown) daily count of clinical reports.

By definition of the aggregation, we have

$$A(\tau(k)) = \sum_{j=\tau(k-1)}^{\tau(k)} y(j) \tag{S1}$$

We want to calculate the variables y such that they are piecewise linear between each aggregation time $\tau(k)$ and continuous. Hence, we have

$$y(\tau(k)+j) = y(\tau(k)) + j \times m_k \tag{S2}$$

for $0 \leq j \leq \tau(k+1) - \tau(k)$. We want to calculate the slope m_k between the aggretation times.

For the beginning of the time series, we set $\tau(0) = 0$ and $A(0) = y(0) = 0$. For this initial period, we have $y(j) = y(0) + j \times m_0$, hence using Equation S2

$$A(\tau(1)) = \sum_{j=1}^{\tau(1)} j \times m_0 = m_0 \frac{(\tau(1)+1)\tau(1)}{2} \tag{S3}$$

That is, rearranging the equation above:

$$m_0 = \frac{2A(\tau(1))}{(\tau(1)+1)\tau(1)} \tag{S4}$$

For the following periods, we have for $k \geq 1$:

$$A(\tau(k+1)) = \sum_{j=1}^{\tau(k+1)-\tau(k)} y(\tau(k)+j) \tag{S5}$$

$$= \sum_{j=1}^{\tau(k+1)-\tau(k)} y(\tau(k)) + j \times m_k \tag{S6}$$

$$= (\tau(k+1)-\tau(k))y(\tau(k)) + \frac{(\tau(k+1)-\tau(k))(\tau(k+1)-\tau(k)+1)m_k}{2} \tag{S7}$$

Rearranging, and setting $\Delta_k = \tau(k+1) - \tau(k)$, we obtain the slope

$$m_k = 2 \times \frac{A(\tau(k+1)) - y(\tau(k)\,\Delta_k)}{\Delta_k(1+\Delta_k)} \tag{S8}$$

This method for interpolating aggregate case reports is specified using the argument `prm.daily = list(method = "linear")` in the function `ern::estimate_R_cl()`.
