# Supplementary material for: ern: An R package to estimate the effective reproduction number using clinical and wastewater surveillance data
Source: PLoS One. 2024 Jun 21;19(6):e0305550. doi: 10.1371/journal.pone.0305550 (PMC11192340; doi:10.1371/journal.pone.0305550)
Supplement: S4 File — (PDF) [file pone.0305550.s004.pdf]

# SUPPLEMENTARY MATERIAL - S4

# ern: an R package to estimate the effective reproduction number using clinical and wastewater surveillance data

David Champredon[1*], Irena Papst[1], Warsame Yusuf[1]

**1** Public Health Risk Sciences Division, Public Health Agency of Canada, National Microbiology Laboratory, Guelph, Ontario, Canada

* david.champredon@canada.ca

**Sensitivity analysis to selected parameters**

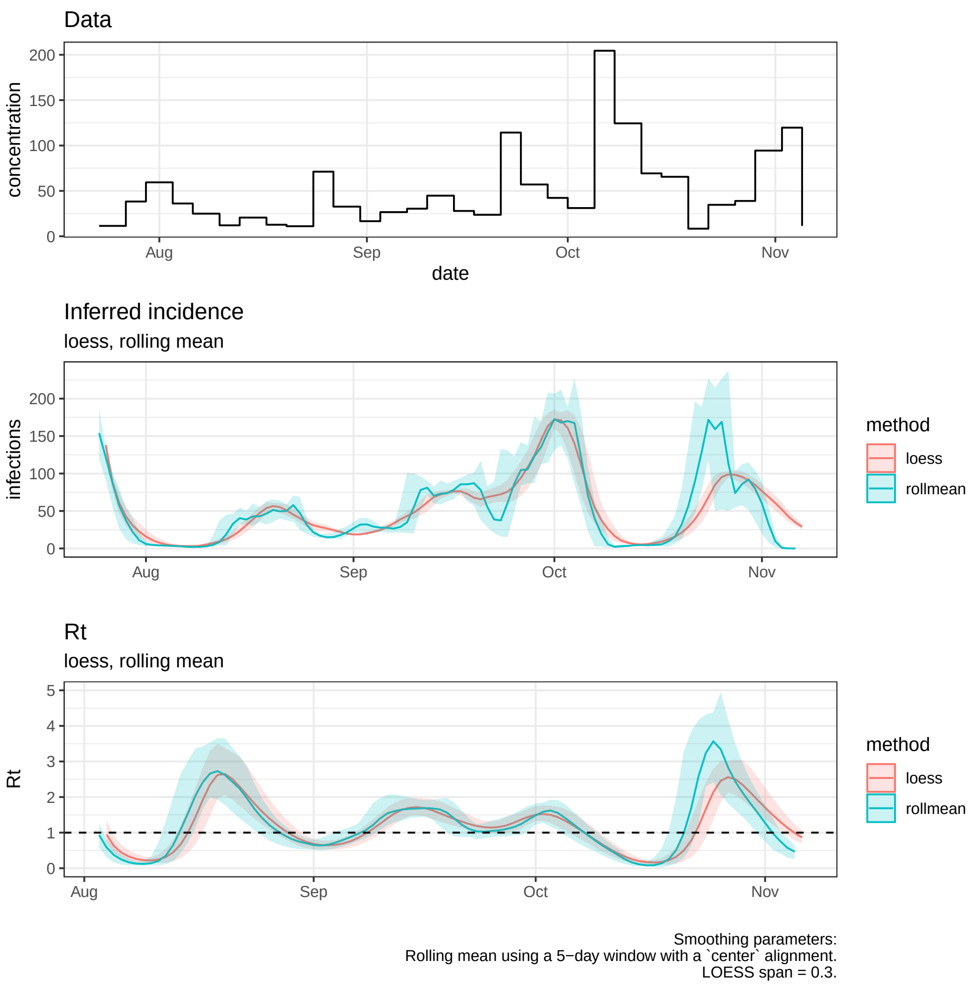


**Fig S4-1. Impact of the smoothing algorithm on the estimation of $\mathcal{R}_t$.** Top panel: viral concentration example data set that ships with the R package `ern`. Middle panel: infered daily latent incidence using LOESS with the span parameter at 0.3, or a 5-day rolling mean. Bottom panel: estimated $\mathcal{R}_t$ after latent daily incidence has been smoothed with LOESS or rolling average. Both smoothing algorithms yield similar $\mathcal{R}_t$ estimates.

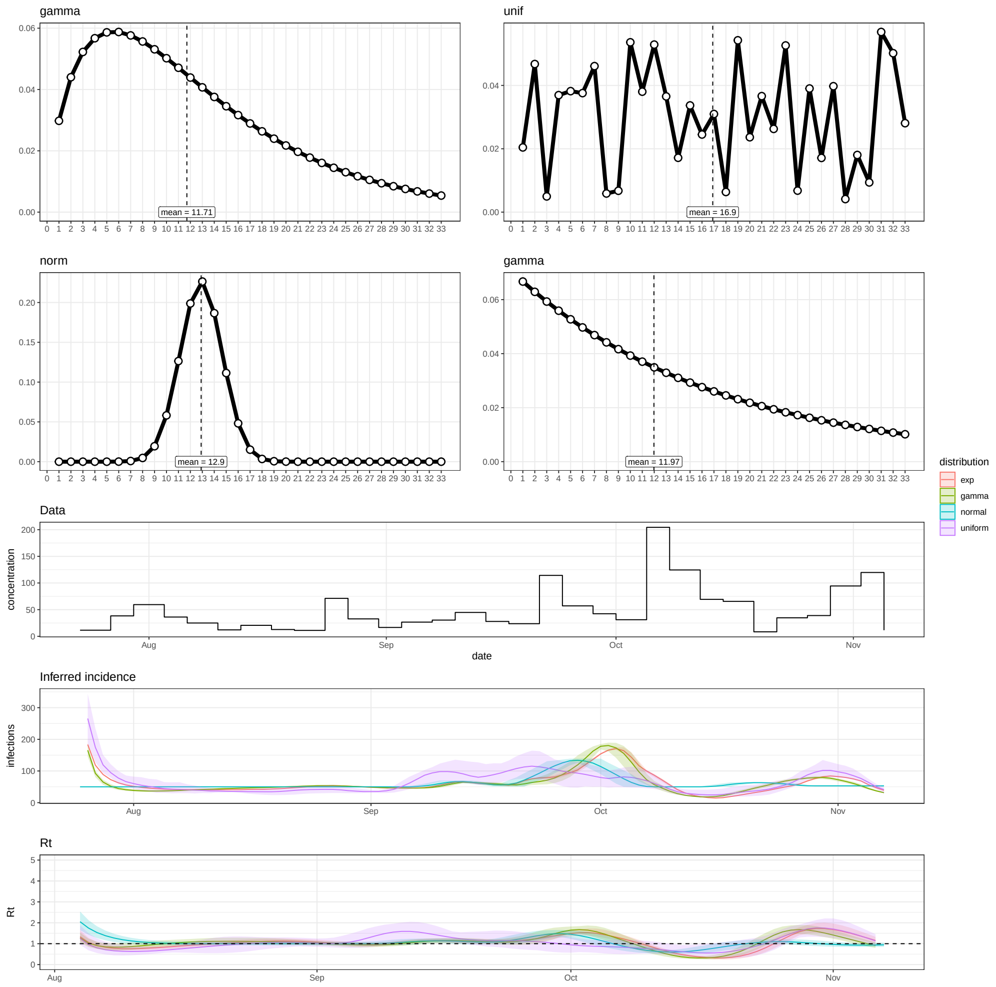


**Fig S4-2. Impact of the choice of the fecal shedding temporal variation on the estimation of $\mathcal{R}_t$.** Top two rows: fecal shedding distributions assumed in this illustration. Third row: viral concentration example data set that ships with the R package `ern`. Fourth row: inferred daily latent incidence using the different temporal distributions for fecal shedding. Fifth row: estimated $\mathcal{R}_t$ using the different temporal distributions for fecal shedding.

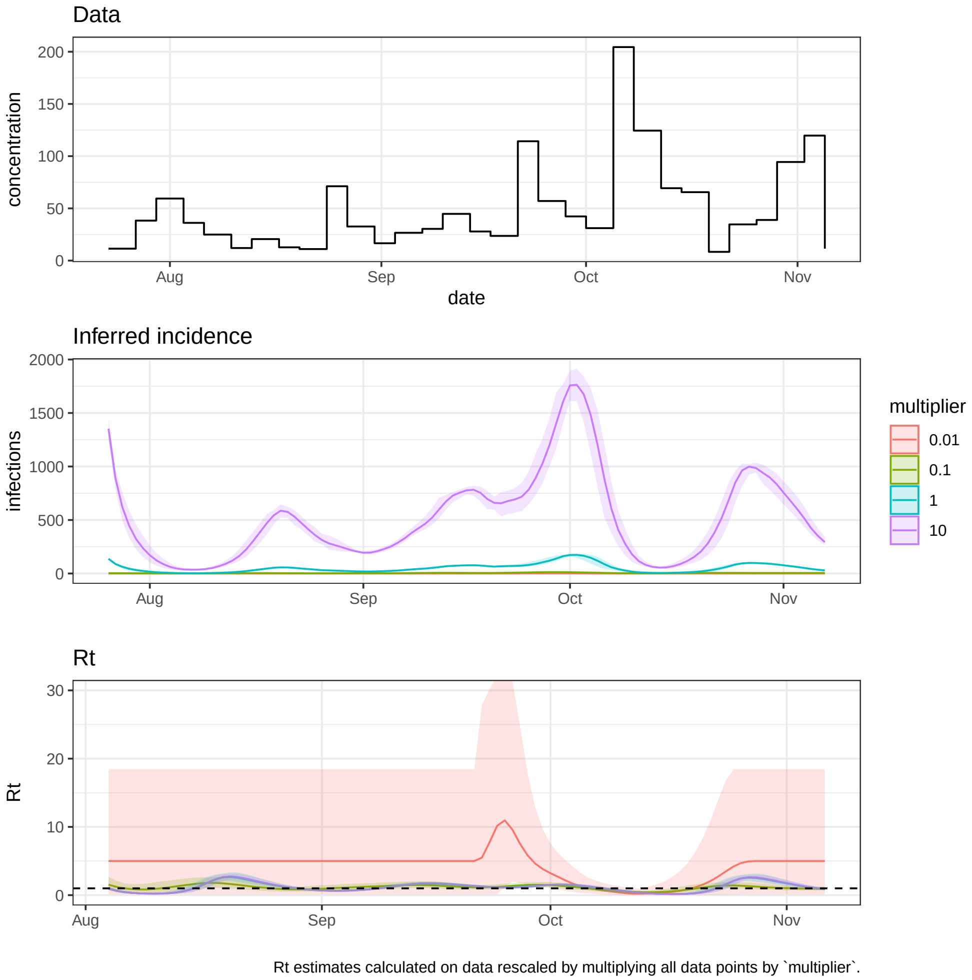


**Fig S4-3. Impact of the level of disease prevalence on the estimation of $\mathcal{R}_t$.** Top panel: viral concentration example data set that ships with the R package `ern`. Middle panel: infered daily latent incidence for different values of the data multiplier (original data when the multiplier value is 1, data values divided by 10 when the multiplier value is 0.1, and so on). Bottom panel: estimated $\mathcal{R}_t$ using different values for the data multiplier.
