# Supplementary material for: ern: An R package to estimate the effective reproduction number using clinical and wastewater surveillance data
Source: PLoS One. 2024 Jun 21;19(6):e0305550. doi: 10.1371/journal.pone.0305550 (PMC11192340; doi:10.1371/journal.pone.0305550)
Supplement: Attachment — Submitted filename: Review.pdf [file pone.0305550.s008.pdf]

REVIEW for submission SMCA-23-02-0390 to IEEE Transactions on Systems, Man and Cybernetics: Systems

"Descriptor Multi-Affine Representation of Rational Uncertain Systems"

by Dongdong Zhao, Lei Yan, Li Xu, and Shi Yan

The authors presented an R software package, which implements statistical methods to estimate the actual number of new infections using the number of reported cases or the wastewater data. It is important to note that the package allows the input data to be sampled by a period higher then one day (e.g., aggregated weekly data is also acceptable). Still, the output is a daily time series, which allows to estimate the effective reproduction number using the already existing tool, EpiEstim (Cori et al. 2013). To estimate the hidden time-series (i.e., the unknown input) from the measured output, the Authors applied a deconvolution using an existing Richardson-Lucy implementation. As far as I know, this technique is equivalent to a dynamic inversion (Silverman 1969 and Isidori 1999, Sec. 5.6), which was already used to infer the effective reproduction number R_t (Csutak et al. 2023).

In the abstract, the authors very diplomatically note the lack of publicly available ***user-friendly*** statistical tools to ***easily*** estimate R_t from wastewater data. I have looked through the software package myself, I have also tried it out, and I agree that it is user-friendly:

- It requires a so-to-say lightweight and free programming environment, R (in contrast to a MATLAB package).
- It requires only a few 3rd party packages, like `EpiEstim`, `assertthat`, `dplyr`, `tidyr`, `lubridate`, `patchwork`, `rjags`, therefore, it can be easily installed. (This was the first time I used R.)
- Example data are provided alongside the code, hence, the results from the manuscript can be easily reproduced.
- The code itself is nicely organized and well-parameterized.

However, I should note that I found a few – possibly not user-friendly, but publicly available – software tools to estimate the effective reproduction number (or at least the incidence) from **wastewater data** possibly in combination with the hospital load or reported cases. (I did not try them out myself):

- Proverbio et al. 2022 have developed a MATLAB package, called the *CoWWAn* (COVID-19 Wastewater Analyser), which makes it possible to infer the shedding population and estimate the effective reproduction number. CoWWAn is available at `https://gitlab.lcsb.uni.lu/SCG/cowwan`. (See also Panel **a** of Fig. 2.)
- Polcz et al. 2023 presented an epidemic reconstruction method, which makes use of the hospital load and/or the wastewater data. A MATLAB implementation is available at `https://github.com/ppolcz/WBE-monitoring-for-COVID-19`.
- McMahan et al. 2021 presented a method to estimate the infected population. The R code is available at `https://github.com/scwatson812/COVID19WastewaterModel`.
- Fazli et al. 2021 proposed a technique to reconstruct the actual number of new cases from the clinical reported cases and/or wastewater data. The R code is available at `https://github.com/Shakeri-Lab/COVID-SEIR`.

Not to mention the **deconvolution-based method** and the associated **R code** developed by Huisman et al. 2022 (referred to as "[14]"), which are publicly available at `https://github.com/JSHuisman/wastewaterRe`. The Authors also observed that their result *"is similar to the one taken in [14]"* (Lines 107–112). However, the Authors did not mention the real advantages of their software over [14] other than "user-friendliness".

Minor comments

- Fig 1. In upper-left block I read "concerntration".
- At Line 116: an noisy.
- Function LOESS at Line 123 is not introduced, first, it seemed to be an abbreviation, then, after a short googling, I realized it is quite standard in statistics especially in R. I think it would be useful to add a reference here, e.g., the documentation page of LOESS (https://www.rdocumentation.org/packages/stats/versions/3.6.2/to
- Line 251, "`plot(dist.fec)`" $\Longrightarrow$ `plot_dist(dist.fec)` (?).
- Line 261, "for the for the". Maybe, an automatic spell-check and grammar check will improve the quality of the text.
- I liked the idea that the parameters of the fecal shedding and symptom generation distributions were also considered as probability variables, and were sampled accordingly.

Overall impression and evaluation.

If I read the manuscript like a user's manual, it's nicely written. Although I cannot detect any scientific contribution in this manuscript, the "attached" R package may be useful for a certain community (e.g., public health practitioners). The manuscript has therefore a raison d'être, possibly not in such a high impact journal (but the Editor is the final judge on that).

Anyway, the Authors need to be better justify why their software tool is preferable or more convenient compared to other existing R/Matlab/Python packages.

References

Cori, A., N. M. Ferguson, C. Fraser, and S. Cauchemez (2013). A new framework and software to estimate time-varying reproduction numbers during epidemics. *American Journal of Epidemiology*, 178(9):1505–1512. DOI: 10.1093/aje/kwt133.

Silverman, L. M. J. P. (1969). Inversion of multivariable linear systems. *IEEE Transactions on Automatic Control*, 14(3):270–276.

Isidori, A. (1999). *Nonlinear control systems II*. London, UK, UK: Springer-Verlag London. DOI: 10.1007/978-1-4471-0549-7.

Csutak, B., K. M. Jenei, and G. Szederkényi (June 2023). Linearization based robust reference tracking control of a compartmental epidemiological model. In: *24th International Conference on Process Control*. Slovakia.

Proverbio, D., F. Kemp, S. Magni, L. Ogorzaly, H.-M. Cauchie, J. Gonçalves, A. Skupin, and A. Aalto (June 2022). Model-based assessment of COVID-19 epidemic dynamics by wastewater analysis. *Science of The Total Environment*, 827:154235. DOI: 10.1016/j.scitotenv.2022.154235.

Polcz, P. et al. (May 2023). Wastewater-based modeling, reconstruction, and prediction for COVID-19 outbreaks in Hungary caused by highly immune evasive variants. *Water Research*, 241:120098. DOI: 10.1016/j.watres.2023.120098.

McMahan, C. S. et al. (Dec. 2021). COVID-19 wastewater epidemiology: a model to estimate infected populations. *The Lancet Planetary Health*, 5(12):e874–e881. DOI: 10.1016/s2542-5196(21)00230-8.

Fazli, M., S. Sklar, M. D. Porter, B. A. French, and H. Shakeri (Dec. 2021). Wastewater-based epidemiological modeling for continuous surveillance of COVID-19 outbreak. In: *2021 IEEE International Conference on Big Data (Big Data)*. IEEE. DOI: 10.1109/bigdata52589.2021.9671543.

Huisman, J. S. et al. (May 2022). Wastewater-based estimation of the effective reproductive number of sars-cov-2. *Environmental Health Perspectives*, 130(5). DOI: 10.1289/ehp10050.
