# Supplementary material for: ern: An R package to estimate the effective reproduction number using clinical and wastewater surveillance data
Source: PLoS One. 2024 Jun 21;19(6):e0305550. doi: 10.1371/journal.pone.0305550 (PMC11192340; doi:10.1371/journal.pone.0305550)
Supplement: Attachment — Submitted filename: PLOS-responses-to-reviewers.pdf [file pone.0305550.s009.pdf]

Response to reviewers

We would like to thank all reviewers for their time and insightful comments that certainly contributed to improving this manuscript and the accompanying software.

Reviewer 1

please abstract must be more clear

Response: We have clarified the abstract based on this comment and comments from other reviewers

material and methods not Models and software 93

Response: We have updated the subsection heading in accordance with this comment.

discussion must be written more advanced reference with more knowledge about your artcle

Response: The Discussion has been improved based on the comments given during this round of reviews and additional references have been inserted.

conculsion must be written with detail

Response: The conclusion paragraph has been improved based on the comments given during this round of reviews.

Reviewer 2

The authors present the R package ern for the estimation of the effective reproduction number from wastewater or aggregated clinical surveillance data. The package provides a framework for an efficient and quicker estimation of effective reproduction number using a user-friendly interface.

The manuscript is well-written and makes a relevant contribution to the field.
I thoroughly enjoyed reviewing this manuscript and only have some minor requests for revision, as follows:

Lines 39 to 42: Do not start a sentence using a citation number. In Line 39, you may write, "Huisman et al. [14] proposed a method....". Do the same for lines 40, 41 and 42.

Response: Changed as suggested.

Line 261: "...for the for the ...". Delete the repetition.

Response: Fixed, thank you.

Reviewer 3

1- It would have been better to talk about the Rt factor, which was mentioned in the research, in numerical terms, with something simple in the abstract

Response: This suggestion has been implemented, thank you.

2 - It was possible to dispense with some paragraphs in figure or table in the introduction

Response: We have added two tables and modified Figure 1 in response to comments from this round of reviews.

3 - The researcher did not provide a research review of references that address the same topic, even in a simple way

Response: A review of references was already provided in the first submission, but we augmented it with more references and contextual comments.

4- It is possible to clarify the work algorithm in the form of clear points or in an algorithmic form, on the basis of which the steps of the example are clarified

Response: We have clarified Figure 1 (that illustrated the overall algorithm) and edited the Materials and Methods section to improve the description of the main algorithm. The supplementary materials have also been improved to illustrate methodologically different approaches to this problem.

5- The discussion was narrative and did not clarify any future idea or plan of action for researchers working in the future in the same field and what difficulties they may face.

Response: We have expanded the Discussion section to include limitations of the current and how they inform planned future developments.

Reviewer 4

1. Describe dataset features in more details and its total size and size of (train/test) as a table.
Response: We have provided more details on the data sets shipped with the package and used for illustrations in the manuscript.

2. Pseudocode / Flowchart and algorithm steps need to be inserted.
Response: We have improved Figure 1 to show a clearer flow chart of the algorithm.

3. Time spent need to be measured in the experimental results.

Response: We have added Table 2 to show the computation times of sample calculations using our package and other comparable R packages.

4. Limitation and Discussion Sections need to be inserted.

Response: There was already a Discussion section where we discussed the various limitations of our approach. However, we have expanded this section using comments we received during this round of reviews.

5. The parameters used for the analysis must be provided in table

Response: We understand this reviewer refers to the input parameters of the functions implemented in the package ern. We do not think that presenting these parameters in a table is necessary. We are not presenting an analysis but showing an illustration of the software, and all the parameters are already clearly shown in the examples and discussed throughout the manuscript. Moreover, as our package is open source, all parameters coded into the software are readily available in the official R package repository CRAN.

6. The architecture of the proposed model must be provided

Response: We have clarified Figure 1 (a flowchart illustrating the overall algorithm implemented in our package) and we have edited the Materials and Methods section to improve the description of the main algorithm. The supplementary materials have also been improved to clarify the methodological different approaches.

7. Address the accuracy/improvement percentages in the abstract and in the conclusion sections, as well as the significance of these results.

Response: It is not possible to compute accuracy/improvement percentages for the quantity our package estimates, Rt, as it is not observable. We did perform comparisons with other packages, but as stated in our manuscript, a thorough comparison between existing packages is beyond the scope of this article.

8. The authors need to make a clear proofread to avoid grammatical mistakes and typo errors.

Response: We have proofread the manuscript and believe it is now free of typos and grammatical errors.

9. Add future work in last section (conclusion) (if any)

Response: Future work was already present in the manuscript submitted, but we have further expanded it based on the comments received during this round of reviews.

10. To improve the Related Work and Introduction sections authors are recommended to review this highly related research work paper:
a) Optimizing epileptic seizure recognition performance with feature scaling and dropout layers
b) Optimizing classification of diseases through language model analysis of symptoms
c) Predicting female pelvic tilt and lumbar angle using machine learning in case of urinary incontinence and sexual dysfunction
d) Utilizing convolutional neural networks to classify monkeypox skin lesions

e) Hepatitis C Virus prediction based on machine learning framework: a real-world case study in Egypt

Response: Thank you for these suggestions. However, we believe none of the articles above are relevant to our study, though we have added several new references based on various comments received during this round of reviews.

Reviewer 5

I would like to thank the authors for putting together the R package ern and for submitting this accompanying manuscript. I agree with the assessment that there is a need for user-friendly statistical software to estimate reproduction numbers from wastewater concentration measurements. This manuscript explains the motivation for developing the package ern, i.e. to provide a dedicated interface to estimate Rt from wastewater and clinical case data. It then describes the statistical approach used and presents a vignette-style illustration of the main functionalities of the package. The authors also propose a new approach to disaggregate non-daily case counts for subsequent Rt estimation.

I have read the manuscript in detail and I have tested the package both using the example data from Canada as provided by the authors, and using wastewater data from Switzerland. I tried to structure my review into manuscript-related and package/method-related major comments, plus a collection of minor comments.

For transparency, I am a (co)author of two packages mentioned in my review, i.e. the package "estimateR" and the package "EpiSewer".

Kind regards
Adrian Lison

Response: Thank you very much for your thorough and thoughtful review. We believe that your comments truly improved our manuscript.

Major points manuscript

First let me say that I found the manuscript clean and well-written.

Related work

I think the manuscript can provide more details on what is methodologically novel and what not. You mention that the method implemented is similar to the method by Huisman et al., but aside from the approach to disaggregate non-daily case data, it seems at first glance to be EXACTLY the method by Huisman et al., i.e. as detailed in https://doi.org/10.7554/eLife.71345 for case data and in https://doi.org/10.1289/EHP10050 for wastewater data (LOESS smoothing, deconvolution using Richardson-Lucy algorithm, scaling, R estimation using EpiEstim, uncertainty quantification via resampling). I am raising this not only because of attribution but because it is important to clearly describe the differences and similarities between related methods such that readers can compare them properly.

Response: Thank you for raising this important point. We fully agree that transparency on the methodology is important. Indeed, we do leverage existing work to build a user-friendly package that merges various existing methodologies for components of our package's Rt estimation. We have tried to clarify this at several points in the revised manuscript (Introduction and Discussion).

Furthermore, there are several related works that are worth mentioning in my opinion.

First, the method by Huisman et al. has also been implemented as an open-source R package (https://doi.org/10.1186/s12859-023-05428-4) "estimateR", and can similarly be used to estimate Rt from wastewater and case data (see e.g. https://ibz-shiny.ethz.ch/wastewaterRe/). Compared to the package ern, the interface and plotting functionality of estimateR are not explicitly tailored to wastewater data, therefore I think that ern is more user-friendly for this domain. Also, estimateR offers no option to disaggregate non-daily case counts. Aside from that, I think that estimateR and ern are highly similar since they are based on almost the same method.

Response: We have added these references in the Introduction, when presenting the existing R packages.

Aside from ern, there are also other R packages for modeling wastewater data, including the package EpiSewer (https://zenodo.org/doi/10.5281/zenodo.10569101), your own package wem (https://github.com/phac-nml-phrsd/wem/tree/main), and the Covid19 Wastewater Analysis Package (https://github.com/UW-Madison-DSI/Covid19Wastewater/tree/main), although the latter does not produce Rt estimates. I do not think a detailed comparison or benchmarking of these packages is necessary, but a short discussion of their differences would be useful. I believe it is valuable to give potential users an overview over available options.

Response: Thank you for making us aware of these works. We have included `EpiSewer` in the comparison section. Because `Covid19 Wastewater Analysis Package` does not provide Rt estimates, we decided to not include it nor any other such tools that support epidemic data manipulation in the comparison with other software. We have, however, added references to compartmental models (including the `wem` package) in the background section. Moreover, we have added Table 1 that provides a summary of the features for similar R packages discussed in the Introduction section.

Sensitivity analyses

I liked the illustrations of the package, but what I missed are some sensitivity analyses that give users an idea of what behavior to expect in different situations and what limitations the method might have. Some interesting analyses that I can think of would for example be about the smoothing (what role do the hyperparameters play, how do moving average and LOESS compare, how does the interpolation deal with larger gaps of data), or what happens if the fecal shedding distribution is misspecified, or how the method performs when concentrations are low. I know that such kinds of analyses require some work, but I think having some sensitivity analyses would add much value to the paper. You can then also point to these analyses from the package documentation.

Response: We have added a section to the manuscript as well as several appendices with the suggested sensitivity analyses.

Limitations

Lines 22f: I agree that wastewater has several advantages over clinical data and does not have the same biases, but it would be good to shortly mention also potential biases of wastewater. In particular I am worried that the statement "Fecal shedding occurs passively and irrespective of the symptomatic status of the infected individual" could be misunderstood by readers. While it is true that asymptomatic patients also seem to shed into wastewater, there is also large variation in shedding loads and distributions between patients and it is not yet clear what the main factors are (see e.g. https://doi.org/10.1016/j.scitotenv.2020.141364 and https://doi.org/10.1128/msphere.00132-23). Wastewater concentrations could also be to a large part be driven by "supershedders" and we don't know how representative this subgroup is of the overall population. Other sources of bias worth mentioning are changing populations in the catchments and environmental factors like rainfall.

Response: Thank you for this suggestion. We have edited the controversial sentences and added a few references. We have also added text describing potential issues with using wastewater concentration data to calculate Rt in the Discussion.

Scaling factor: Can you give some more details on how you would choose this in practice, and what the implications of a potential misspecification are (for example that a constant factor of misspecification will strongly bias incidence estimates but at least not bias Rt except in certain edge cases).

Response: We added some details about using a scaling factor in practice in the Discussion.

For the above reason, I would stress in the manuscript (and also in the package documentation) that the inferred incidence directly depends on the hard-to-estimate scaling factor and must therefore be carefully interpreted. Otherwise there is a risk that people will use this to estimate prevalence etc.

Response: Thank you for this suggestion, we have added a warning regarding the interpretation of the incidence implied from the wastewater data in the Discussion.

I suggest to add unit information to the concentration and scaling factor. For example, what are the typical units of the exemplary data in ww.input?

Response: We have added this information in the manuscript ("Example with wastewater data" section) as well as in the R package documentation.

Disaggregation

The proposed method for disaggregation of non-daily cases and the comparison with the method by Nash et al. is quite interesting. Based on your illustration, it seems like the method by Nash et al. could have important limitations not found in the original study by Nash et al.. Since this would be a strong result, can you provide more details, e.g. which

sliding window was used in the example? also plot Rt estimated from the daily incidence time series?

Response: Our goal was not to highlight limitations of other packages but rather warn readers that different methods can lead to different inferences. Doing a thorough comparison of the different methods is beyond the scope of this manuscript. We have changed the Supplementary material S1 in that sense and provided more details, as suggested.

In your model for inferring daily case counts, you do not seem to account for potential changes in transmission other than due to susceptible depletion. What happens if you fit this over longer time periods with multiple waves or time series with strong non-pharmaceutical interventions?

Response: Yes, this is a limitation of this method (for prm.daily(method="renewal")) and will be addressed in future versions of the package. In our experience, this is satisfactory for single waves where no major interventions interfering with disease spread (for example seasonal influenza). In the meantime, we have added a new method based on linear interpolation to the package (argument prm.daily(method="linear")) that can handle those types of trajectories (e.g., multiple waves, strong NPI). We have added some text in the Discussion to reflect these changes.

Lines 379f: I think this is a rather problematic approach - drawing not enough posterior samples and then applying smoothing to improve the irregular posterior. I think this can easily lead to an unrepresentative posterior and also distort the uncertainty estimates.

Response: Thank you for pointing this out. We have added MCMC diagnostic outputs to the R package and a warning when the Gelman-Rubin R statistic is above 1.025. Moreover, we added text to draw the reader's attention about the potential pitfalls of not using enough iterations (like for any MCMC-based algorithm).

In the introduction, you mention long runtimes of epidemia and EpiNow2 as disadvantages to overcome, but the disaggregation of ern also requires MCMC sampling. How do the runtimes compare on non-daily data, is ern still considerably faster?

Response: We have added Table 2 that compares the computing times between packages.

Major points package / method

The package ern currently only seems to offer a fixed scaling factor. Explicitly supporting a time-varying scaling factor to account for flow would be great. Scaling concentrations by daily flow volumes at the treatment plant can be quite important in my experience because there can be a strong effect of dilution of the viral particles by rainfall etc. on the measured concentration.

Response: Indeed, a time-varying scaling factor can have multiple uses, including the integration of varying shedding profiles (from different lineages, for example, as stated in a previous comment) and also to integrate "metadata" like wastewater flow. Thanks again for making this suggestion, we plan

to implement a time-varying scaling factor in the near future.

Lines 173: What output of EpiEstim is used for Rt? Do you use the estimated mean of Rt? Or do you draw samples from the posterior Gamma distribution estimated by EpiEstim?

Response: The way in which EpiEstim is used within ern to estimate Rt is more clearly explained in the revised Materials and methods section. We hope the answer to the above question is now clear from the manuscript.

I noticed that the Rt estimates provided by ern do not have higher uncertainty towards the present, although this should definitely be the case (Rt of today cannot be estimated with the same accuracy as Rt of last week because of delays in fecal shedding / reporting). Do you account for uncertainty in the deconvolution step?

Response: Unlike the `EpiSewer` framework, the model in `ern` does not have the latent incidence as a random variable when estimating from wastewater data, so this uncertainty is not accounted for. The uncertainty of the fecal shedding distribution is propagated but, as such, that does not lead to a higher uncertainty closer to the date of calculation. We did not have the time to fully explore this interesting question for this round of review but will investigate this more deeply as we continue to develop our package.

I think it is a great feature that ern also supports uncertain distributions. One question I had is if you could also support correlated parameters. At the moment, the mean and sd of a distribution get drawn independently, but they may be correlated in reality. Another scenario that may be quite realistic is that users have several different distributions / parameters from the literature. In this case you could allow users to provide a list of distributions from which to draw with replacement. These are just suggestions, not requests for this paper.

Response: Thank you for this suggestion. Correlated parameters for uncertain distributions is not currently a feature of our package, but we will consider adding this feature in a future version.

I am still a bit unsure about the default distributions provided in the package. On the one hand, this is a practical feature, but on the other hand: how are you planning to maintain/update this epidemiological data? They seem to be hard-coded in the package, so if newer distributions become available or old distributions are invalidated by new research, users will only get the updates if they install a newer version of the package. It would be good to comment on this.

I am particularly skeptical of providing default reporting delay distributions or reporting proportions as they will differ a lot between surveillance systems / countries etc.

I currently find it hard to define custom distributions with my own parameters. Functions like def_dist_incubation_period only accept the name of a pathogen as input. I know I can also construct a list with custom parameters myself, but a constructor function with the relevant parameters would be helpful.

There is an argument for subtypes/variants in def_dist_fecal_shedding, but it does not seem to do anything, I always get back the same distribution.

Also, def_dist_reporting_fraction does not accept a custom value and assumes a uniform distribution between 0.1 and 0.3 per default. This seems quite arbitrary to me.

Response: We agree with the points raised about issues with providing default distributions. We have removed pre-defined distributions from our package. The user must now define all distributions themselves, and we have provided a constructor function "def_dist()" for this purpose.

I like the diagnostic plots, but having the option to produce individual plots for concentration, incidence, Rt etc. would be valuable. They are currently all merged into one plot via the patchwork package, making it hard to customize individual plots.

Response: This is now implemented, thank you for the suggestion.

Minor points

Line 5: "It differs from the basic reproduction number, R0, in that it takes into account the level of susceptibility in the population at a given point in time." Maybe say more generally that it accounts for "changes in transmission" - this may be due to changing susceptibility or other factors like different contact patterns, infection control measures etc.

Response: Updated in response to this suggestion.

Lines 21f: I would also mention digital droplet PCR as an alternative quantification method. Also, maybe shortly mention that viral RNA is first extracted from the wastewater sample using various laboratory methods.

Response: Changed as suggested.

Line 78: EpiNow2 and epidemia do not use the rstan package, they use cmdstanr package. I would just write "stan".

Response: Changed as suggested.

Lines 113: This is an important point and well explained.

Response: Thank you.

Lines 166f: This paragraph felt a bit informal and was difficult to understand. Is your main point that it is important to estimate Rt using the number of cases by time of infection, not by time of report / time of sample?

Response: We have changed the text and hopefully clarified this paragraph.

I personally don't find the name of the function ern::ww.input very intuitive, I would not have expected that this returns example data! Also, can you provide more details in the function documentation, e.g. what the column "pt" means and what the unit of the concentration is in this example?

Response: We have changed the names of the data set shipped with the package and have provided more details of the sample data via the package documentation.

Lines 137f, reporting delay: I would say more clearly that this is the delay between symptom onset and case report.

Response: Changed as suggested.

As a suggestion, you could register the current version of the package in an online archive like zenodo. This will give you a DOI for the package, which can then be referenced in the manuscript.

Response: Thank you. Between the time of submission of this manuscript and this round of reviews, we successfully submitted the `ern` package to CRAN. This is the reference we are now using in this manuscript.

References

1. Huisman, J. S. _et al._ Estimation and worldwide monitoring of the effective reproductive number of SARS-CoV-2. _eLife_ **11**, e71345 (2022).
2. Huisman, J. S. _et al._ Wastewater-Based Estimation of the Effective Reproductive Number of SARS-CoV-2. _Environmental Health Perspectives_ **130**, 057011 (2022).
3. Scire, J. _et al._ estimateR: an R package to estimate and monitor the effective reproductive number. _BMC Bioinformatics_ **24**, 310 (2023).
4. Lison, A. EpiSewer: Estimate Reproduction Numbers from Wastewater Measurements. Zenodo (2024).
5. Jones, D. L. _et al._ Shedding of SARS-CoV-2 in feces and urine and its potential role in person-to-person transmission and the environment-based spread of COVID-19. _Science of The Total Environment_ **749**, 141364 (2020).
6. Arts, P. J. _et al._ Longitudinal and quantitative fecal shedding dynamics of SARS-CoV-2, pepper mild mottle virus, and crAssphage. _mSphere_ **8**, e00132-23 (2023).
7. Nash, R. K., Bhatt, S., Cori, A. & Nouvellet, P. Estimating the epidemic reproduction number from temporally aggregated incidence data: A statistical modelling approach and software tool. _PLOS Computational Biology_ **19**, e1011439 (2023).

Reviewer 6

The authors presented an R software package, which implements statistical methods to estimate the actual number of new infections using the number of reported cases or the wastewater data. It is important to note that the package allows the input data to be sampled by a period higher then one day (e.g., aggregated weekly data is also acceptable). Still, the output is a daily time series, which allows to estimate the effective reproduction number using the already existing tool, EpiEstim. To estimate the hidden time-series (i.e., the unknown input) from the measured output, the Authors applied a deconvolution using an existing Richardson-Lucy implementation. As far as I know, this technique is equivalent to a dynamic inversion, which was already used to infer the effective reproduction number.

Although I cannot detect any scientific contribution in this manuscript, the ``attached'' R package may be useful for a certain community (e.g., public health practitioners), and the software description in the main body is clear and didactic.
The manuscript has therefore a raison d'être, possibly not in such a high impact journal (but the Editor is the final judge on that).

Anyway, the Authors need to be better justify why their software tool is preferable or more convenient compared to other existing packages.

Response: For this round of reviews, we have made multiple edits to highlight some benefits in using the package "ern", including compared to other similar packages. We hope they address this comment.

The authors presented an R software package, which implements statistical methods to estimate the actual number of new infections using the number of reported cases or the wastewater data. It is important to note that the package allows the input data to be sampled by a period higher then one day (e.g., aggregated weekly data is also acceptable). Still, the output is a daily time series, which allows to estimate the effective reproduction number using the already existing tool, EpiEstim (Cori et al. 2013). To estimate the hidden time-series (i.e., the unknown input) from the measured output, the Authors applied a deconvolution using an existing Richardson-Lucy implementation. As far as I know, this technique is equivalent to a dynamic inversion (Silverman 1969 and Isidori 1999, Sec. 5.6), which was already used to infer the effective reproduction number R_t (Csutak et al. 2023).
In the abstract, the authors very diplomatically note the **lack** of publicly available ***user-friendly*** statistical tools to ***easily*** estimate R_t from wastewater data. I have looked through the software package myself, I have also tried it out, and I agree that it is user-friendly:

- It requires a so-to-say lightweight and free programming environment, R (in contrast to a MATLAB package).
- It requires only a few 3rd party packages, like EpiEstim, assertthat, dplyr, tidyr, lubridate, patchwork, rjags, therefore, it can be easily installed. (This was the first time I used R.)
- Example data are provided alongside the code, hence, the results from the manuscript can be easily reproduced.
- The code itself is nicely organized and well-parameterized.

However, I should note that I found a few – possibly not user-friendly, but publicly available – software tools to estimate the effective reproduction number (or at least the incidence) from **wastewater data** possibly in combination with the hospital load or reported cases. (I did not try them out myself):

- Proverbio et al. 2022 have developed a MATLAB package, called the *CoWWAn* (COVID-19 Wastewater Analyser), which makes it possible to infer the shedding population and estimate the effective reproduction number. CoWWAn is available at https://gitlab.lcsb.uni.lu/SCG/cowwan.

(See also Panel **a** of Fig. 2.)

- Polcz et al. 2023 presented an epidemic reconstruction method, which makes use of the hospital load and/or the wastewater data. A MATLAB implementation is available at https://github.com/ppolcz/WBE-monitoring-for-COVID-19

Response: Thank you for highlighting these two MATLAB packages. However, in this manuscript we focus only on packages written in the programming language R.

- McMahan et al. 2021 presented a method to estimate the infected population. The R code is available at https://github.com/scwatson812/COVID19WastewaterModel.
- Fazli et al. 2021 proposed a technique to reconstruct the actual number of new cases from the clinical reported cases and/or wastewater data. The R code is available at https://github.com/Shakeri-Lab/COVID-SEIR.

Response: Thank you for highlighting these two references. The listed GitHub repos were created to support published papers but the R code has not been developed to provide a generic tool to estimate the epidemiological parameter R_t. We have, however, added these references in the background discussion featured in the Introduction of our manuscript.

Not to mention the **deconvolution-based method** and the associated **R code** developed by Huisman et al. 2022 (referred to as "[14]"), which are publicly available at https://github.com/JSHuisman/wastewaterRe. The Authors also observed that their result *"is similar to the one taken in [14]"* (Lines 107–112). However, the Authors did not mention the real advantages of their software over [14] other than "user-friendliness".

Response: We have addressed this comment when responding to similar comments from another reviewer.

Minor comments

• Fig 1. In upper-left block I read "concerntration".

Response: Fixed, thank you.

• At Line 116: an noisy.

Response: Fixed, thank you.

• Function LOESS at Line 123 is not introduced, first, it seemed to be an abbreviation, then, after a short googling, I realized it is quite standard in statistics especially in R. I think it would be useful to add a reference here, e.g., the documentation page of LOESS (https://www.rdocumentation.org/packages/stats/versions/3.6.2/to

Response: Thank you for this suggestion, we have added text to explain the LOESS algorithm in brief and added the seminal paper (Cleveland, 1979) as a reference.

• Line 251, "plot(dist.fec)" =⇒ plot dist(dist.fec) (?).

Response: Corrected.

• Line 261, “for the for the”. Maybe, an automatic spell-check and grammar check will improve the quality of the text.

Response: Corrected.

• I liked the idea that the parameters of the fecal shedding and symptom generation distributions were also considered as probability variables, and were sampled accordingly.

Response: Thank you.

Overall impression and evaluation.
If I read the manuscript like a user’s manual, it’s nicely written. Although I cannot detect any scientific contribution in this manuscript, the “attached” R package may be useful for a certain community (e.g., public health practitioners). The manuscript has therefore a raison d’ˆetre, possibly not in such a high impact journal (but the Editor is the final judge on that).
Anyway, the Authors need to be better justify why their software tool is preferable or more convenient compared to other existing R/Matlab/Python packages.

Response: We believe the scientific contribution from “ern” is to provide a tool that empowers the scientific community to use and interpret easily wastewater-based epidemiological surveillance data and also to make meaningful inferences from standard clinical surveillance, often reported weekly. We have added a short clarification in the Discussion section, and hope the various edits addressing all reviewers’ comments make our contribution clearer.
