# Supplementary material for: ern: An R package to estimate the effective reproduction number using clinical and wastewater surveillance data
Source: PLoS One. 2024 Jun 21;19(6):e0305550. doi: 10.1371/journal.pone.0305550 (PMC11192340; doi:10.1371/journal.pone.0305550)
Supplement: Attachment — Submitted filename: Response to Reviewers - round 2.docx [file pone.0305550.s010.docx]

Response to Reviewers – Round 2

We would like to thank the reviewers for their time to read and comment on our revised manuscript.

In response to Reviewer #5’s comments:

Thank you for the thorough revision and for addressing the comments. I have two remaining remarks, which however do not necessitate another review round in my opinion.

First, thank you for answering my question with regard to accounting for uncertainty of the Rt estimates towards the present. I understand the current deconvolution method is not able to account for this uncertainty. But I think it is very important to highlight this limitation more strongly in the manuscript, i.e. in the discussion, but also to make users aware of this in the package documentation etc. Otherwise, the use of the package could lead to wrong conclusions when using it for real-time surveillance. The uncertainty towards the present because of partial information is an inherent characteristic of the data and means that we can only get accurate Rt estimates with a certain delay. If this is not reflected in the uncertainty information provided by the method, users should be made aware of this limitation.

*Response: Thank you for this suggestion. We have added a paragraph in the Discussion section to reflect this limitation.*

Second, you describe that to pool the uncertainty of the Rt estimates, you get the mean and quantiles for each realization and then compute means or quantiles of these across realizations. This seems rather approximate to me, and I wonder why you don't just draw e.g. 100 Rt samples from the posterior Gamma distribution as described by Cori et al. (you can use the estimated mean and cv from EpiEstim) for each realization, then combine all the draws across your realizations and compute the mean, empirical quantiles etc. on this pooled posterior sample. This should also be very fast and more accurate.

*Response: Thank you very much for this suggestion. Indeed, the way this was implemented was not statistically correct (we were not aware of the EpiEstim function “sample_posterior_R”). It is now implemented as suggested (*[*link*](https://github.com/phac-nml-phrsd/ern/commit/95bc6b68c3457357cd580b2bad312a0772d70167)*). A few tests showed that the numerical difference between both implementations was very small. The main text has been edited to reflect this change.*
